# Does Open Reduction in Intramedullary Nailing of Femur Shaft Fractures Adversely Affect the Outcome? A Retrospective Study

**DOI:** 10.1155/2020/7583204

**Published:** 2020-05-20

**Authors:** Syed Imran Ghouri, Abduljabbar Alhammoud, Mohammed Mubarak Alkhayarin

**Affiliations:** Hamad Medical Corporation, Doha, Qatar

## Abstract

**Aim:**

This study aims to assess the results of open versus closed reduction in intramedullary nailing for femoral fractures and whether it delays union, predisposes to nonunion, or increases the rate of infection.

**Materials and Methods:**

A retrospective review of all adult patients with isolated femoral shaft fractures treated by intramedullary nailing was done. The primary outcome is union rate, and the secondary outcomes are operation time and the infection rate.

**Results:**

110 isolated femoral shaft fractures, with 73 (66.4%) in the closed reduction group and 37 (33.6%) in the open reduction group, 90.4% males and 9.6% females, and the average age was 32.6 years. RTA is the most common cause of these injuries followed by the fall from height. The delayed union rate was 20% (22/110) with no difference between the two groups, *p* value 0.480, and the nonunion rate was 5.5% (6/110), and no statistical difference was observed between the two groups. The operation time was shorter in the closed groups, and no difference in the time to union was observed between two groups. No infection was found in the two groups.

**Conclusions:**

There is no statistical difference between the healing rates in closed and open reduction in femoral shaft fractures. In cases where closed reduction is difficult, it is better to open reduce the fracture if closed reduction cannot be achieved in 15 minutes, especially in polytrauma.

## 1. Introduction

Fractures of the femoral shaft are due to high energy trauma and therefore can be associated with life-threatening injuries and causes of permanent disability. Intramedullary nailing is the standard of care for the management of femoral shaft fractures in adults with union rates between 95 and 99% [1]. Though the complication such as nonunion and malunion is still a challenge in such fracture especially in subtrochantric, pediatrics age group, and floating knee, this technique can be done with either closed (without disruption of the fracture site with indirect reduction) or open reduction (through small incision over the fracture with direct reduction) [2]. Remarkable improvements in the operative treatment of these injuries in the last 15 years have dramatically lessened the morbidity and mortality associated with these fractures [3]. Closed locked intramedullary nailing is now the management of choice in femoral diaphyseal fractures. However, closed reduction may not always be achievable, and the only option then is to open the fracture site to achieve an acceptable reduction. This is an additional trauma to the patient and alters the biology of the fracture.

The aim of this study to ascertain if open reduction during intramedullary nailing of femoral shaft fractures is detrimental to fracture healing, operating times, and infection rates comparing to the closed one.

## 2. Materials and Methods

A retrospective review of all adult patients with isolated femoral shaft fractures treated by intramedullary nailing at level one trauma center between 2011 and 2015 was done after obtaining the ethical approval from Medical Research Center.

Patients with isolated closed, diaphyseal femur shaft fracture were included, whereas those with fractures of the proximal or distal femur treated with other modalities, open fractures, head injury and polytrauma, inadequate data availability, and nonavailability of follow-up were excluded.

Data were collected for general demographic (age and gender), injury characteristic (mechanism of injury and fracture classification), and outcome finding (union rate, infection rate, secondary procedure, and operation time).

Delayed union was considered when no bridging callus was seen at 6 months after surgery as per standard FDA definition, whereas nonunion was established when no bridging callus was seen on radiographs at 12 months after surgery [4].

All patients were operated in the lateral decubitus position using statically locked AO Synthes femoral nails, and in cases with open reduction, an additional incision was made over the fracture site, and with one or two fingers, the reduction and rotation were checked.

Descriptive statistics were used to summarize demographic data and injury characteristics. We used a chi-squared test and a Fisher exact test to express the associations between two or more qualitative data points, whereas an unpaired *t*-test was used to compare the quantitative data between the two groups. Frequency (percentage) and mean ± SD or median and range were used for categorical and continuous values as appropriate. A *p* value of <0.05 was considered statistically significant. All statistical analyses were done using statistical packages SPSS 23.0 (SPSS Inc., Chicago, IL) and Epi InfoTM 2000 (Centers for Disease Control and Prevention, Atlanta, GA).

## 3. Results

110 adult patients with isolated femoral shaft fractures treated by intramedullary nailing were included in the study, and 73 (66.4%) underwent closed reduction and 37 (33.6%) required open reduction and subsequent insertion of a femoral nail.

### 3.1. Demographic

**Figure 1:**
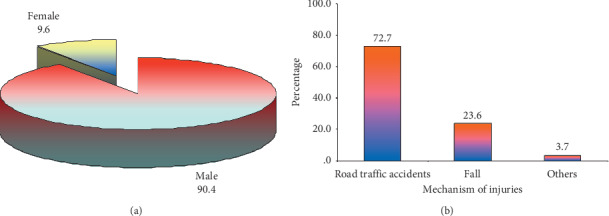
(a) Gender; (b) mechanism of injuries.

Out of a total of 110 patients, 90.4% were males and 9.1% were females with 62 males and 5 females in the closed reduction group and 32 males and 5 females in the open reduction group (Table 1 and Figure 1).

The mean age of the patients in the closed reduction group was 31.6 years and in the open reduction group was 33.08 years.

### 3.2. Injury Characteristic

Mechanism of injury in most of patients in our series was victims of road traffic accidents with head on or side impact injuries; others sustained falls, especially the laborers working on construction sites (Figure 1 and Table 1).

We adopted the Winquist and Hansen classification for this study for fracture classification. Eighty fractures were Winquist type 1 (53 in the closed reduction group and 27 in the open reduction group). Twenty-four were Winquist type 2 (16 in the closed reduction group and 8 in the open reduction group). Three fractures were Winquist type 3 (2 in the close group and one in the open group). Three fractures were segmental (2 in the closed reduction group and 1 in the open reduction group) (Table 1).

### 3.3. Bone Healing Outcome

The union was delayed in 22 (20%) patients, comprising 16 cases in the closed reduction and 6 in the open reduction group, and no statistical difference in delay union between two groups was observed, *p* value (0.480).

**Figure 2:**
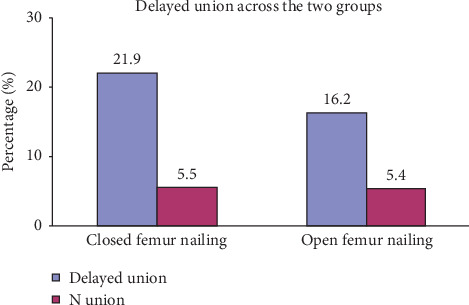
Delayed union and no union.

Six patients had nonunion of the fracture with 4 nonunions in the closed reduction group (5.5%) and 2 in the open reduction group (5.4%). The *p* value again was not significant. All the nonunion patients were managed by secondary autogenous bone grafting, and union was achieved in all cases (Table 2 and Figure 2).

The mean time to union in the closed reduction group was 7.11 months with a standard deviation of 3.496. The mean time to union in the open reduction group was 7.35 months with a standard deviation of 4.673. The *p* value here again was not significant.

### 3.4. Other Outcomes

The mean operating time in the closed reduction group was 113.2 minutes with a standard deviation of 34.725. The mean operating time in the open group was 132 minutes with a standard deviation of 35.670. The *p* value was significant. However, we believe that the longer operating time in the open group was probably due to the complex nature of these fractures to start with. No patient in our series developed any superficial or deep infection.

## 4. Discussion

This study aims to find out whether open reduction with subsequent drainage of the fracture hematoma and the additional tissue trauma affects the union and rehabilitation with more complication rates in femoral shaft fractures when compared with the closed reduction technique which is the gold standard of management of these injuries [2, 3].

That being said, open intramedullary nailing of the femur does have certain advantages like using less expensive equipment than that required for closed nailing; no special fracture table is required; image intensifier is not (or briefly) required; or absolute anatomical reduction is easier to obtain than with closed means [5]. Direct observation of the bone may identify undisplaced and undetected comminution not noted radiographically which can be dealt with. Precise interdigitation of the fracture fragments improves rotational stability. In segmental fractures, the middle segment can be stabilized, preventing torquing and twisting associated with closed reduction and medullary reaming. In nonunions, opening of the medullary canals of sclerotic bones is easier, and rotational malalignment is rare after open reduction.

Some disadvantages of the open technique have also been described which include the consideration of skin scars, loss of fracture hematoma which is important for fracture healing, and bone shavings obtained from reaming the canal are lost. Infection rates are increased, union rates are decreased, and image intensification may still be required if a locking nail is used [6].

Because it requires no special equipment and achieves quick reduction, some authors advocate open nailing in the polytrauma patients [5]. Open intramedullary nailing is invaluable in the first trimester pregnant polytrauma patient with least radiation exposure [7].

Grundnes et al. in their study on open versus closed femur nailing in rats concluded that the fractures did heal faster initially with closed nailing, but at 12 weeks, there was no significant difference in the mechanical characteristics [8]. Furthermore, some studies actually showed judicious use of open reduction techniques during intramedullary nailing of closed fractures which appeared to have a minimal risk of infection [7–9]. Our study has shown that the overall risk of nonunion or infection is unchanged in both types of reduction. Wolinsky et al. demonstrated a union rate of 93.6% after initial nailing and an overall union rate of 98.9% following an additional procedure [10]. Leighton et al. also showed 97% satisfactory results with open nailing as compared to 92% with closed nailing [11]. Closed reamed intramedullary nailing technique is still the preferred method and has a greater chance of healing and lower rate of complications [12–16]. However, there are still controversies in results of femoral shaft fractures treated by close versus open nailing [17–20]. This study also reveals the fact that a well-timed and proper open reduction of a femoral fracture during nailing does not impede the healing or eventual functional outcome of the fracture, and the incidence of infection is similar. We did not record intraoperative radiation exposure in both groups by dosimetry; this may be considered as a relative weak point of the study along with the relatively small sample size.

One important factor that may impact in the outcome of femur fracture is using the locking screws as using the locking screws leads to increase surgical time, blood loss, and radiation exposure without significant impact in the fracture healing.

Dealing with nonunion and malunion is challenging in femur fracture, and proper and systematic follow-up is a key to deal with any delay union which can be managed by dynamization which leads to optimal results in such fractures.

Retrograde nailing is considered a good option for antegrade nailing in treatment of femur fracture with almost similar results with regard to the functional and radiological outcomes.

Open reduction and intramedullary nailing of femoral shaft fractures did not significantly increase delayed union or nonunion rates or predispose to infection. It only resulted in a longer operative time which was probably due to the complexity of the injury itself and can be considered a safe alternative to closed reduction in situations where closed reduction cannot be obtained and or in polytrauma patients.

The operating surgeon ought to be prepared to open the fracture if a satisfactory closed reduction cannot be attained within a reasonable interval of operating time. The potential benefits for the patient outweigh the theoretical pitfalls of this additional procedure. This, in our study, did not increase the risk of reducing the functional result. A prospective study in this regard will perhaps shed more light on the topic.

## Figures and Tables

**Table 1:** Demographic data.

	Total	Closed group	Open group	*p* value
Number	110	73 (66.4%)	37 (33.6%)	
Gender				0.316
Male	94 (90.4%)	62 (92.5%)	32 (86.5%)	
Female	10 (9.6%)	5 (7.5%)	5 (13.5%)	
Age (year)		31.16 + 11.04	33.08 + 14.62	0.453
Mechanism of injury				0.696
RTA	80 (74.8%)	53 (75.7%)	27 (73.0%)	
Fall	26 (24.3%)	16 (22.9%)	10 (27.0%)	
Others	1 (0.9%)	1 (0.9%)	0 (0%)	
Fracture classification:				1.0
Type 1	80 (72.7%)	53 (72.6%)	27 (73.0%)	
Type 2	24 (21.8%)	16 (21.9%)	8 (21.7%)	
Type 3	3 (2.7%)	2 (1.8%(	1 (0.9%)	
Type 4	3 (2.7%)	2 (1.8%)	1 (0.9%)	

**Table 2:** Outcome data.

	Total	Closed group	Open group	*p* value
Delay union	22 (20%)	16 (21.9%)	6 (16.2%)	0.480
Nonunion	6 (5.5%)	4 (5.5%)	2 (5.4%)	0.987
Time to union (months)		7.111 + 3.4	7.3 + 4.6	0.802
Operation time (minutes)		113 + 34.7	132 + 35.6	0.010
Infection rate	0	0	0	

## Data Availability

The data used to support the findings of this study are available from the corresponding author upon request.
